# Is It Time to Reconsider the Lipopolysaccharide Paradigm in Acute Graft-Versus-Host Disease?

**DOI:** 10.3389/fimmu.2017.00952

**Published:** 2017-08-09

**Authors:** Etienne Daguindau, Thomas Gautier, Cécile Chagué, Jean-Paul Pais de Barros, Valérie Deckert, Laurent Lagrost, Philippe Saas

**Affiliations:** ^1^Université Bourgogne Franche-Comté, EFS Bourgogne Franche-Comté, INSERM, UMR1098, Besançon, France; ^2^Department of Hematology, University Hospital of Besançon, Besançon, France; ^3^LipSTIC LabEx, FHU INCREASE, Besançon, France; ^4^INSERM, Université Bourgogne Franche-Comté, LNC UMR1231, Dijon, France; ^5^LipSTIC LabEx, Dijon, France; ^6^University Hospital of Dijon, Dijon, France

**Keywords:** graft-versus-host disease, endotoxin, lipopolysaccharide, TLR4, PLTP, lipoproteins, microbiota, Escherichia coli

## Introduction

The curative efficiency of allogeneic hematopoietic cell transplantation (HCT) is considerably dampened by the graft-*versus*-host disease (GVHD) that leads to significant mortality and morbidity. Donor T cell subsets are recognized as the main cellular mediators and effectors of acute GVHD (aGVHD). T cell interactions with antigen-presenting cells (APC) of both host and donor origin are required to achieve the status of “alloreactive activated T cell” generating the cytotoxic attack against target organs. However, a preliminary awakening of APC by exogenous or endogenous alarm signals from distressed/injured cells is critical to recruit and drive an efficient T cell activation. Endogenous signals are known to be released after the conditioning regimen (1). They consist in “damage-associated molecular patterns” (DAMP) that are not discussed here. Exogenous signals are a group of widespread natural microbial patterns called “pathogen-associated molecular patterns” (PAMP) and are supposed to be translocated from the microbiota in case of body natural barrier weakness, especially gut mucosa. We will focus here on the role of a major component of the Gram-negative bacteria, the lipopolysaccharide (LPS), which is one of the most studied PAMP in immunology and in the aGVHD pathophysiology.

## The Role of LPS in GVHD Initiation

Both the conditioning regimen (chemotherapy and/or total body irradiation) and the subsequent tissue injuries induced by GVHD compromise gastrointestinal tract integrity. This promotes LPS translocation in the systemic circulation. Hill and colleagues have first highlighted the role of conditioning regimen in LPS leakage in the blood circulation and demonstrated that higher dose of irradiation in recipient mice correlated with more lethal GVHD. In this work, highly irradiated mice showed enhanced pro-inflammatory cytokine (TNF-α and IL1-β) production associated with higher LPS blood concentrations (2). The cellular damages induced in the gut during GVHD have been assessed. Intestinal stem cells and Paneth cells are implicated in the regeneration of the intestinal epithelium and the production of antimicrobial peptides (AMP), respectively. These cell subsets were identified as primarily targets of gut GVHD in experimental models (3, 4), inducing loss of gut integrity. Moreover, inhibition of AMP production by Paneth cells favored outgrowth of *Escherichia coli* in the gut flora, which represents the major source of LPS. This creates a vicious circle due to the pro-inflammatory role of LPS (4). Finally, electron microscopy analysis of colon lesions allowed direct visualization of tight junction impairment during aGVHD, which leads to paracellular permeability (5, 6). Further studies identified the importance of gut barrier integrity to avoid PAMP leakage. Thus, IL-23 has been shown to have a significant impact on colonic inflammation during GVHD. In an experimental model using donor IL-23-deficient APC, reduction of gut damages was demonstrated along with marked reduction of circulating LPS (7). In human setting, a Phase I/II randomized study addressed the prevention of mucositis by palifermin (keratinocyte growth factor) administrated after HCT in order to limit LPS translocation. Even though a reduced severity of mucositis was shown, the study failed to demonstrate significant effect on GVHD occurrence/severity (8).

After transplant, LPS is considered as a systemic mediator that binds to the toll-like receptor 4 (TLR-4)/myeloid differentiation factor 2 (MD2) complex at the surface of innate immune cells in GVHD target organs such as skin, liver, and gut. This interaction amplifies the secretion of pro-inflammatory cytokines (i.e., IL-1β, TNF-α, and IL-6) in a process called “cytokine storm” (9). Another effect of the LPS binding on TLR-4 expressed by APC is the upregulation of major histocompatibility complex and costimulatory molecule expression on the APC surface. When the TLR-4 signaling pathway is inhibited either with a TLR-4 antagonist administrated to recipient mice (10) or in recipient mice deficient in TLR-4 (11), the severity of aGVHD decreases significantly. This suggests the importance of LPS in GVHD mechanisms. However, TLR-4 is the receptor for other ligands, including both PAMP and DAMP (12).

Considering the microbiota as the unique source of LPS and its key role in GVHD pathophysiology, one can easily suggest that the beneficial effect on aGVHD of gut decontamination in recipient mice demonstrated by van Bekkum et al. (13) is presumably due to reduced LPS translocation. Nowadays, this assumption needs to be integrated with recent identifications of the wide interactions between intestinal epithelium, gut flora, and the immune system based on new molecular tools (14).

## Innovative Insights on LPS to Reconsider “Former” Data

Most informative data published on LPS structure and functions have been issued from *E. coli*-derived LPS. LPSs are amphipathic molecules comprising three chemically distinct regions: the O-antigen, the core oligosaccharide, and the lipid A. The lipid A consists in a variable amount of fatty acid chains (mostly from three to six). This hydrophobic moiety binds to TLR-4, and therefore, carries the inflammatory properties mostly demonstrated during infections with Gram-negative bacteria and in endotoxin shock (15, 16). TLR-4 triggering by LPS transduces a downstream pathway implicating signal adaptor proteins, MyD88 and TRIF. We highlighted the critical role of the phospholipid transfer protein (PLTP), a member of the lipid transfer/LPS binding protein (LT/LBP) family, in LPS metabolism and beyond in sepsis (17). Because PLTP mediates the transfer of a spectrum of plasma lipids and the antioxidant tocopherol on lipoproteins (18), PLTP was first found to be involved in lipid homeostasis and also in atherosclerosis. Among the amphipathic molecules affected by the transfer properties of PLTP, LPS has been identified (19). This specific function displayed by PLTP allows scavenging of LPS from the circulation to biliary secretion and has been called the “reverse LPS transport.” It has been demonstrated *in vitro* a lower production of pro-inflammatory cytokines in LPS-stimulated splenocytes from wild-type (WT) mice compared to those of splenocytes from PLTP-deficient (PLTP^−/−^) mice in the presence of lipoproteins as “LPS-carrier.” This experiment sustained the role of PLTP in LPS metabolism. The “reverse LPS transport” was also addressed in the context of endotoxin shock, and more recently in the setting of sepsis: PLTP^−/−^ mice exhibited a significant higher mortality rate than WT mice (17, 20). Thus, proteins from the LT/LBP family should be considered in the GVHD pathophysiology.

The liver is the main organ involved in LPS clearance. Recent data demonstrated that liver sinusoidal endothelial cells are faster LPS scavenger than Küpffer cells (21). In this work, the “LPS-carrier” function of high-density lipoprotein (HDL) is shown to provide a protective role in inflammatory responses by enhancing liver detoxification of LPS. This strengthens the “reverse LPS transport” hypothesis discussed above. Liver dysfunction (i.e., iatrogenic liver diseases, sinusoidal obstruction syndrome, liver infections, or aGVHD) occurs frequently after HCT. These damages might be a cause for defective LPS clearance. Ruutu et al. have shown that there were improved survival and decreased severity of GVHD in patients treated with a hydrophilic bile acid, ursodeoxycholic acid (UDCA) after HCT (22). The reason of this beneficial activity has not been explored so far. It might be interesting to assess the impact of UDCA on LPS elimination by the liver in this setting.

Previous data on the role of LPS in experimental aGVHD pathophysiology are based on the *Limulus amebocyte* lysate (LAL) assay to measure circulating LPS. This LAL assay relies on activation of an enzymatic cascade activated by LPS binding. This assay presents a limitation, since it detects only the biologically active free LPS and is unable to quantify the “neutralized” LPS carried by lipoproteins. An available HPLC/MS/MS method has been set up for the direct quantitation of total amounts of LPS in plasma, encompassing LPS integrated within lipoprotein complexes (23). Briefly, this HPLC-based method can determine concentrations of specific hydroxylated fatty acids from the lipid A. Combined with the LAL assay, this innovative method constitutes a relevant and practical approach to evaluate the ratio of biological active endotoxins and neutralized part of LPS in biological samples. Although the endotoxin activity of LPS is the “visible tip” of the iceberg in GVHD, quantification of whole LPS with the HPLC method associated with the LAL assay gives the opportunity to assess the potency of LPS detoxification by the PLTP in experimental GVHD models and further in transplanted patients. Single nucleotide polymorphisms (SNP) of PLTP have been identified, but not systematically associated with a variation of its function based on HDL concentrations (24, 25). However, these studies did not consider the impact of SNP on the LPS transfer activity of PLTP. This should be explored in the context of HCT: GVHD severity might be predicted by prior assessment of the PLTP capacity to eliminate LPS. Finally, data on “reverse LPS transport” provide a powerful rational to investigate promising strategies of GVHD prophylaxis by modulating PLTP activity to promote LPS elimination.

Previous data on the role of LPS in experimental GVHD pathophysiology assume that LPS measured using the LAL assay is a homogeneous pro-inflammatory entity. To the best of our knowledge, the LPS used experimentally to demonstrate its deleterious impact on GVHD severity is extracted from *E. coli*. We propose to reconsider the assumption that LPS/TLR-4 interactions result in a unique agonist pro-inflammatory reaction in GVHD, since heterogeneity of its sources and structures exist. Indeed, the lipid A structure is highly conserved for a given bacterial species, but varies among the different species regarding the length of fatty acid chains, their number, their acylation, or phosphorylation pattern. The HPLC method mentioned above allows the identification and quantification of different structures of lipid A in biological samples. This heterogeneity in the structure of the lipid A leads to differential effects on the innate immune response (26). A recent study identified the different immunogenicity of LPS according to the variation of the microbiome during the three first early years; this work was initially performed to validate the “hygiene hypothesis” that might explain susceptibility to autoimmune diseases in different populations. The authors found intrinsic strain diversity in microbiome between distinct geographic areas. The LPSs from different main strains were tested *in vitro* by functional assays: LPS extracted from *Bacteroides dorei* was demonstrated to be an inhibitor of the powerful immune stimulation induced by *E. coli*-derived LPS (27). This antagonism between *Bacteroides* LPS and *E. coli* LPS has already been reported several years ago (28). The microbiome and its interaction with immune system are implicated in the occurrence and severity of GVHD. For instance, Jenq and colleagues identified the genus *Blautia* associated with less severe GVHD and non-relapse mortality. This work studied the associations of bacterial genera with GVHD-related mortality in patients by linear discriminant analysis. Interestingly, in their analysis, the *Bacteroides* genus was significantly associated with less GVHD-related mortality (29). Antibiotics are used to treat febrile aplasia after HCT. A greater loss of the *Bacteroidetes* phylum in patient gut microbiome was shown with the piperacillin–tazobactam association compared to aztreonam or cefepime. This piperacillin–tazobactam regimen was associated with a higher rate of GVHD-related mortality (30). The potential causality of less *Bacteroidetes* in patient gut microbiome has not been explored further in this work. One may hypothesize that the LPS of certain microbiota strains (e.g., *Bacteroides*) plays a major role after HCT as a regulator of inflammatory signals delivered by *E. coli*-derived LPS. Considering this links between dysbiosis and GVHD, fecal microbiota transplantation has been recently reported as a promising tool to restore symbiosis and improve steroid refractory GVHD in two cohorts of seven patients (31, 32). It is still unclear how the inoculation of a global “healthy” microbiota can modulate GVHD. So far, the beneficial effect of microbiome-derived metabolites after HCT has been identified for gut epithelium healing (6). According to this model, it would be interesting to assess the source and immunogenicity of microbiota-derived LPS in patients with or without GVHD. The aim would be to identify specific LPS candidates that are associated with better outcome, and then, propose on this identification transplantation of selected bacterial species to prevent or treat GVHD.

## Conclusion

Convincing data on the role of LPS as a major initiator/amplifier of aGVHD have been published so far. However, LPS/TLR-4 interactions have never been established as a specific target translated in human for the treatment or prophylaxis of aGVHD. Reduced intensity conditioning regimens have been hypothesized to limit GVHD incidence through a decrease in inflammation. Another radical approach developed in 1970s was the gut decontamination to suppress bacteria as the source of LPS. This concept has been validated in mice and widely applied in patients. However, gut decontamination was efficiently performed in human, and results of such approach on GVHD occurrence were disappointing. Nowadays, several research axes address the improvement of gut integrity after HCT to limit LPS translocation.

We would like to highlight that a side of LPS physiology has been neglected in GVHD pathophysiology but should deserve attention. First, metabolism of LPS and especially its detoxification by PLTP and lipoproteins have been demonstrated to be critical in sepsis shock. This so-called “reverse LPS transport” seems powerful to modulate endotoxin activity of LPS after HCT and decrease GVHD severity. Furthermore, new tools such as the HPLC-based method are available to explore this hypothesis by quantifying LPS anchored in lipoprotein complexes which are not detected by the classical LAL assays. Second, LPS produced by the whole microbiome is a heterogenic entity with potential opposite activity on innate immune cells. Considering this assumption, a part of the mechanistic link between microbiota and GVHD target organs might be elucidated. We suggest here the beneficial role of *Bacteroides* phylum in GVHD outcome in patients, because through its LPS, it may exert an antagonist inflammatory activity on *E. coli*-derived LPS.

## Author Contributions

ED, TG, CC, J-PPB, VD, LL, and PS critically read, analyzed, and discussed the literature and conceived the outline of the manuscript; ED and PS wrote the manuscript. All the authors edited the manuscript and provided valuable discussions and criticisms.

## Conflict of Interest Statement

The authors declare no competing financial interests, excepted LL and J-PPB that are inventors on a patent application concerning the HPLC/MS/MS methods used to quantify LPS. The reviewer, TS, and handling editor declared their shared affiliation, and the handling editor states that the process nevertheless met the standards of a fair and objective review.
